# Function and Dysfunction of Complement Factor H During Formation of Lipid-Rich Deposits

**DOI:** 10.3389/fimmu.2020.611830

**Published:** 2020-12-08

**Authors:** Seppo Meri, Karita Haapasalo

**Affiliations:** ^1^ Department of Bacteriology and Immunology, University of Helsinki, Helsinki, Finland; ^2^ Department of Bacteriology and Immunology, Translational Immunology Research Program, University of Helsinki, Helsinki, Finland

**Keywords:** apoE, C-reactive protein, adiponectin, HDL, amyloid-beta- protein

## Abstract

Complement-mediated inflammation or dysregulation in lipid metabolism are associated with the pathogenesis of several diseases. These include age-related macular degeneration (AMD), C3 glomerulonephritis (C3GN), dense deposit disease (DDD), atherosclerosis, and Alzheimer’s disease (AD). In all these diseases, formation of characteristic lipid-rich deposits is evident. Here, we will discuss molecular mechanisms whereby dysfunction of complement, and especially of its key regulator factor H, could be involved in lipid accumulation and related inflammation. The genetic associations to factor H polymorphisms, the role of factor H in the resolution of inflammation in lipid-rich deposits, modification of macrophage functions, and complement-mediated clearance of apoptotic and damaged cells indicate that the function of factor H is crucial in limiting inflammation in these diseases.

## Introduction

A major function of the complement system is to handle invading microbes and clear debris, but without sufficient regulation it can attack and destroy our own cells and tissues. It can be activated through three pathways: the classical, alternative, and lectin pathways. The alternative pathway is constantly active in human plasma and responsible for amplifying all the complement activation cascades. To prevent potentially harmful complement attack toward host tissues the amplification pathway of complement is regulated by membrane inhibitors CD35, CD46, and CD55 and by soluble complement factor H and its alternatively spliced product factor H-like protein (FHL-1) (1). Factor H functions not only in the fluid phase to keep complement activation under control but also on surfaces to prevent attack against host targets. FHL-1 lacks the the ability to discriminate between self and nonself targets.

**Figure 1:**
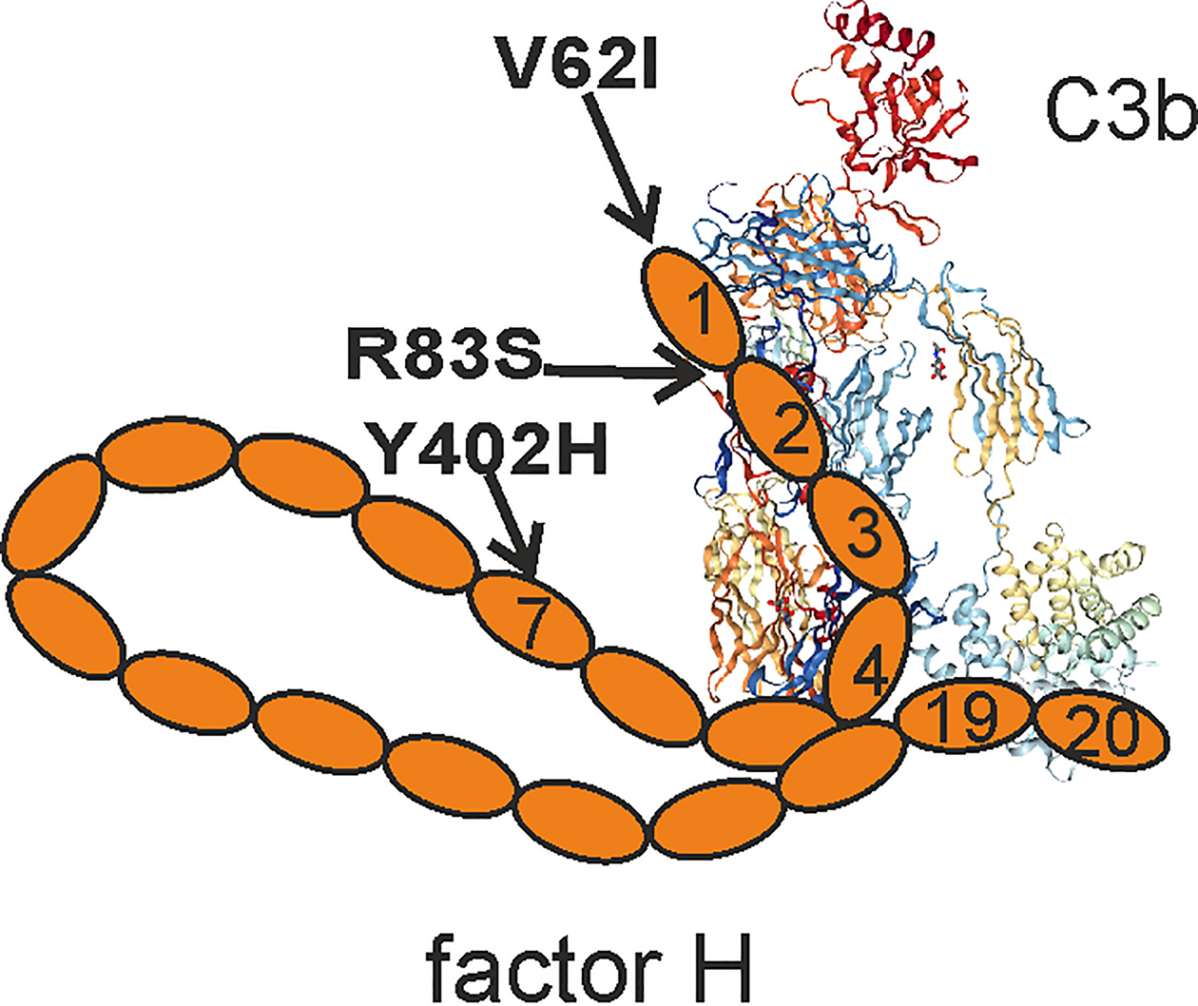
Schematic presentation of factor H domain structure. Factor H domains 1–4 bind to C3b and regulate alternative pathway activation, while domains 19–20 are responsible for surface recognition. The positions of the disease-causing mutations discussed in this manuscript are indicated. C3b crystal structure is from PDB 5FO7.

Factor H recognizes specific host markers directly, such as sialic acids and glycosaminoglycans, or indirectly *via* C-reactive protein (CRP) present or bound on self cell surfaces or apolipopoprotein E (apoE) on high-density lipoprotein particles (2–4). Binding to CRP is mediated *via* domains 6-8 and 19–20 of factor H, while domains 5–7 interact with apoE. Factor H usually binds to these structures in the context with surface-deposited C3b and acts as a cofactor for factor I-mediated inactivation of C3b to iC3b (1). From the 20 domains of factor H, the domains 7 and 19–20 mediate surface recognition, while domains 1-4 are required for regulatory activity (**Figure 1**). Several known mutations and polymorphisms in factor H and anti-factor H antibodies directed against these domains are associated with diseases that can be harmful for the carrier. This indicates that full function of factor H is essential in keeping the spontaneous alternative pathway activation in check and in preventing complement attack against self-structures (5–7).

Due to its ability to bind CRP and control the alternative pathway complement factor H has a central role in the non-inflammatory clearance of extracellular deposits and dying cells in areas of tissue damage (8, 9). Much of the material to be cleared includes various types of phospholipids from cell membranes. Failure in this clearance mechanism may lead to excessive inflammation because of complement activation and overstimulation of macrophages. As a consequence, macrophages can release free radicals that can oxidize lipids and other materials and eventually harm the local tissue. The role of complement is not only restricted to clear microbes and dead cells from human tissues, but it also has an important role in lipid metabolism. For example, complement expression levels are significantly elevated in visceral adipose tissue, and increased expression levels of *CFB* gene have been suggested to associate positively with triglyceride levels and negatively with high-density lipoprotein (HDL) levels in plasmas of obese individuals. This indicates that complement system is involved in the metabolic consequences associated with increased visceral fat mass (10, 11) and in diseases characterized by the presence of lipid-rich deposits.

## Diseases with Lipid-Rich Deposits and Factor H Association

**Figure 2:**
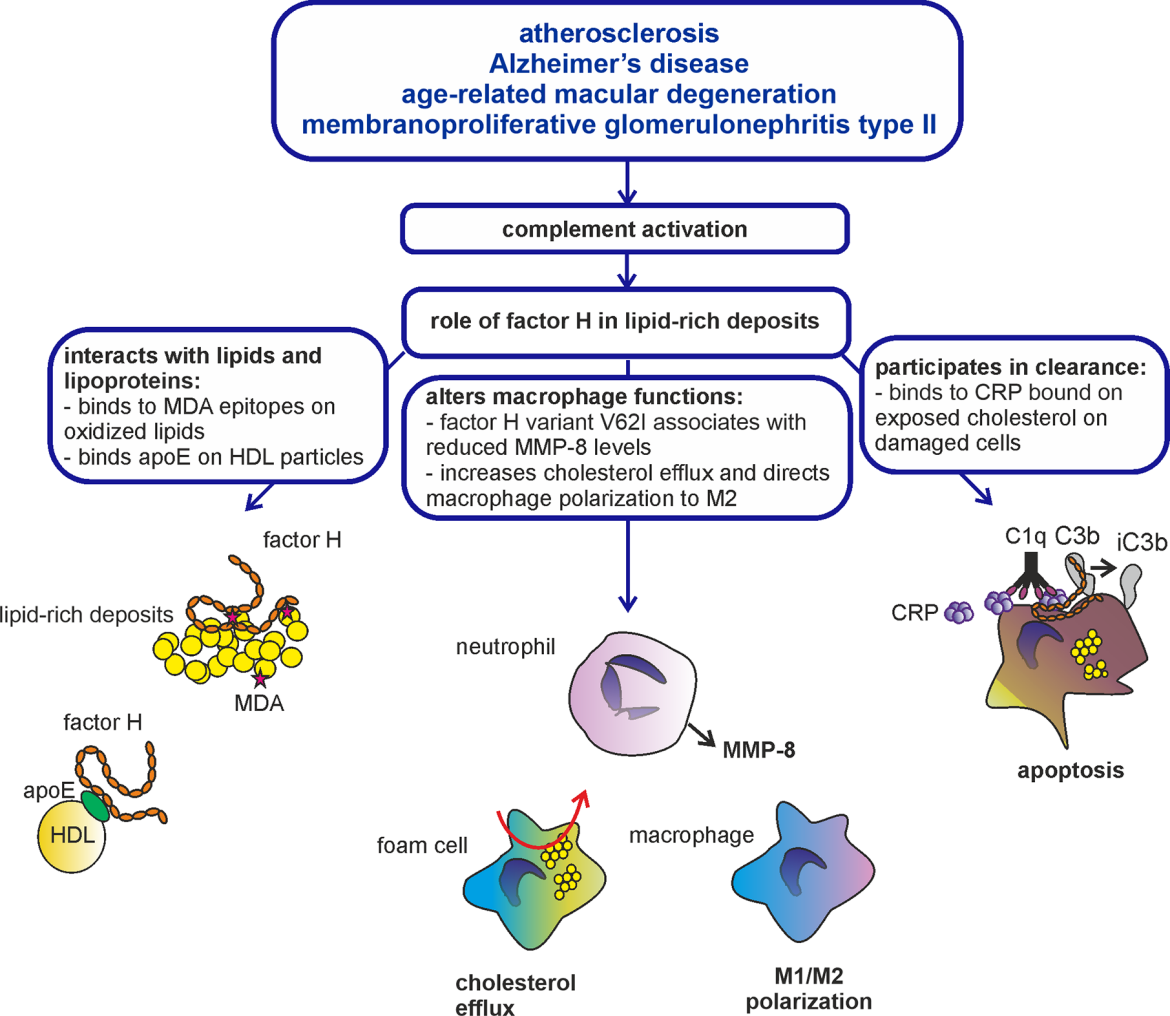
Role of factor H in lipid-rich deposits. Atherosclerosis, Alzheimer’d disease, AMD, and C3G are diseases characterized by formation of lipid-rich deposits and complement-mediated inflammation. Complement regulator factor H is known to bind modified lipids and lipoproteins, interact with macrophages and participate in the clearance of damaged and apoptotic cells through acting as a cofactor for inactivation of C3b to iC3b. Therefore, factor H is likely involved in the resolution of inflammation in lipid-rich deposits accumulated in the arteries, brain, eyes, and kidneys.

Age-related-macular degeneration (AMD), C3 glomerulopathy (C3G) encompassing two different syndromes: C3 glomerulonephritis (C3GN) and dense deposit disease (DDD; previously called membranoproliferative glomerulonephritis type II; MPGN-II), atherosclerosis (AS) and Alzheimer’s disease (AD) have similar histopathological features. They all are associated with accumulation of lipid-rich deposits in the retina (AMD), subendothelially in kidney glomeruli (C3GN), glomerular basement membrane (DDD), arterial intima (AS), or brain (AD). These deposits are called drusen in AMD, dense deposits in DDD, plaques in atherosclerosis, and senile plaques in Alzheimer’s disease. Despite the different names the deposits share common features such as the presence of oxidized lipids and proteins, cholesterol and other lipids and apolipoproteins (12–14) (**Figure 2**). These diseases are also often affecting the same patient. As an example, individuals with DDD often develop ocular drusen in the macula, whose histopathology is indistinguishable from the drusen in AMD (15). Moreover, patients with atherosclerosis are at higher risk to develop Alzheimer’s disease (16) similarly as AMD patients are at higher risk to develop atherosclerosis (17). These findings indicate that the development of the lipid-rich deposits could share a common pathophysiological mechanism. For example, alterations in vascular glycosaminoglycans that bind the complement inhibitor factor H could lead to both lipid accumulation and complement damage in atherosclerotic lesions (18). Finally, these diseases have been studied for their genetic association with the factor H Y402H polymorphism that is located in the domain seven of this molecule. This polymorphism was first found to associate with AMD (19–21). It confers a five-fold increased risk of developing the disease (22). Interestingly, the AMD risk variant 402H also associates with DDD, and possibly also with Alzheimer’s disease and atherosclerosis, although conflicting results on the latter two have been found in different genetic studies (23–27).

### Age-Related Macular Degeneration

Age-related macular degeneration (AMD) is the most common cause of visual loss in the elderly people in industrialized countries. Accumulating evidence suggests that a defect in complement regulation by factor H and other abnormalities in the alternative pathway amplification process play a crucial role in the pathogenesis of the disease. The hallmark of early AMD is formation of drusen between the basal surface of the retinal pigmented epithelium (RPE) and Bruch’s membrane. This leads later to necrosis of the RPE cells. Apart from the the genetic background, aging and smoking are the main risk factors for developing AMD (28).

### Atherosclerosis

Atherosclerosis is a multifactorial disease driven by inflammation and vascular changes. It is caused by accumulation of lipids, immune cells and fibrous elements in the subendothelium of arteries. The thickening and hardening of the arterial wall may lead to a total obstruction of the blood vessel because of plaque rupture. A major critical site is the coronary arteries of myocardium, where a local infarction may occur. Rupture of coronary artery plaques has been shown to be associated with complement activation in myocardial infarction (29). Similarly, atherosclerosis may lead to cerebral infarction or be involved in the rupture of carotid aneurysms (30). The hallmark of early atherosclerotic lesion is the formation of fatty streaks composed of cholesterol-laden macrophages, which are called foam-cells. The foam cells are formed through macrophage-mediated phagocytosis of oxidized and modified low density lipoproteins (LDLs) that accumulate in the subendothelium of arteries. The local inflammation induces differentiation of monocytes into proinflammatory M1 type macrophages that play a crucial role in the pathogenesis of atherosclerotic plaques (31). It seems like the complement system and especially the alternative pathway of complement has a pro-inflammatory role in in atherosclerosis as it serves as an amplification mechanism for C3b generation (32).

### Alzheimer’s Disease

Alzheimer’s disease is characterized by accumulation of amyloid-β, formation of neuronal plaques and neuroinflammation in the brain. Impaired clearance of amyloid-β by microglial cells leads to accumulation of senile plaques in the brain. These can activate the complement system that triggers the development of an inflammatory phenotype in microglia, the phagocytic cells in brain (33). The activated microglial cells trigger astrocytes, which amplify the pro-inflammatory signals and neurotoxic effects (34). Activated microglia are the main source of complement components such as C1q in the brain (35). Interestingly, the classical pathway of complement with its components C1q and C4 appear to contribute to the complement- and microglia-mediated loss of brain synapses in early Alzheimer’s disease (36). Moreover, binding of C1q to apoE has been suggested to reduce C1q-mediated activation of the classical pathway implicating that apoE, the major factor related to Alzheimer’s disease, acts as a complement inhibitor (37). It has, however, been suggested that the alternative pathway of complement could be responsible for the pro-inflammatory effects in the pathogenesis of Alzheimer’s disease (38). Alzheimer’s disease, AMD and atherosclerosis are all degenerative age-dependent diseases, where complement-mediated inflammation most likely plays a crucial role, as several markers of complement activation have been found in the diseased tissues (39, 40).

### C3 Glomerulopathy

C3 glomerulopathy (C3G) entails two different diseases, where complement activation, and especially the alternative pathway, has a central role in disease pathogenesis (41). In DDD, C3b deposits accumulate in the glomerular basement membranes and can be seen as dense deposits by electron microscopy. In C3 glomerulonephritis (C3GN), no dense deposits are seen, but C3b accumulation occurs subendothelially and mesangially. In a proportion of cases, C3GN is associated with immunoglobulin paraproteins (42). In DDD, autoantibodies have been detected against against the C3bBb convertase, so called C3 nephritic factors (43) and against the N-terminus of factor H (6). C3GN and DDD are rare chronic nephritic diseases (44). DDD is more common in children. The inflammation in C3G is mediated *via* hyperactivation of complement that is triggered because of complement dysregulation in the fluid phase. DDD sometimes occurs in association with partial lipodystrophy (PLD). It is characterized by loss of subcutaneous fat from the upper part of the body. It has been suggested that complement-mediated lysis of factor D expressing adipocytes is induced by C3 nephritic factor (C3Nef) in this disease (45). Moreover, in one study, a family with DDD and PLD was found to carry an R83S mutation in factor H in the interface region between domains one and two causing a defect in complement regulation. This indicated a role for alternative pathway dysregulation in the pathogenesis of this disease (46). Several complement components are expressed by adipose tissue including C2, C3, C4, C7, factor B, factor D (D, adipsin), FH, and adiponectin (10, 11, 47, 48). It has been suggested that complement is involved in mediating inflammation in the adipose tissue, but this is maintained only at a reasonable level due to expression of complement regulatory proteins, including factor H (49). Therefore, although not yet shown, a defect in factor H mediated complement regulation could also be involved in the loss of adipose tissue in DDD-related PLD.

## Lipid-Rich Deposits

According to current knowledge, inflammation is the key driver in the formation of lipid-rich deposits in AMD, C3G, atherosclerosis, and Alzheimer’s disease. However, the initial trigger has not yet fully been defined except in rare cases, where carrying a mutation can be directly linked to the disease in the family (46). Conversely, lipid deposits may also undergo changes and modifications that convert them into promoters of inflammation. The particles may become proinflammatory themselves or in the context of their microenvironment.

### Inflammatory Markers

Accumulation of complement components and inflammatory markers such as CRP around lipid-rich deposits indicate that the deposits have triggered inflammation (50–52). It is known, for example, that surface-exposed cholesterol binds CRP that activates the classical pathway of complement and triggers inflammation in the tissue (53). In atherosclerotic lesions CRP binds to phosphocholine in modified low-density lipoproteins (LDL) and colocalizes with LDL in human atherosclerotic lesions (54). In this context factor H plays a crucial role in limiting alternative complement activation through simultaneous binding to CRP and C3b (8). Inactivation of C3b to iC3b promotes noninflammatory clearance of certain lipid particles and dying cells through interaction with CR3 and CR4 receptors on macrophages (9).

In addition to CRP, there are other pentraxins that have been suggested to play a role in the pathogenesis of at least AMD, atherosclerosis, and Alzheimer’s disease. Pentraxin 3 (PTX3) is expressed by RPE cells and glial cells *in vitro* and may be involved in oxidative stress-mediated cell injury (55, 56). Moreover, PTX3 is a marker of disease severity in cardiovascular diseases and may exhibit atheroprotective effects (57, 58). Similarly to CRP, PTX3 opsonizes apoptotic and damaged cells and interacts with factor H (59). The involvement of PTX3 also in C3G is possible, because factor H-related protein 5 (FHR5), which is implicated in DDD and FHR5-related glomerulopathy, enhances complement activation by inhibiting binding of factor H to PTX3 (60).

Matrix metalloproteinase 8 (MMP-8) is a pro-inflammatory marker secreted by neutrophils. Elevated plasma and serum levels of MMP-8 associate with conditions such as peritonitis, rheumatoid arthritis and cardiovascular diseases. A recent study showed a significant association between the V62I polymorphism in domain one of factor H and neutrophil MMP-8 levels suggesting that this variation, with increased regulatory activity, could be involved in suppressing complement activation, MMP-8 expression, and inflammation in cardiovascular diseases (61, 62). Interestingly, this same factor H V62I variant is also associated with decreased susceptibility to AMD (63), further strengthening the hypothesis that the function of factor H is crucial in limiting inflammation in these diseases.

### Danger-Associated Molecular Patterns

Danger-associated molecular patterns (DAMPs) indicate structures that are normally hidden but exposed during tissue injury. These can be recognized by multiple different types of receptors that often recognize also structures found on microbes. The receptors for DAMPs include Toll-like receptors (TLRs) and Nod-like receptors (NLRs) that can lead to activation of the inflammasome structures inside cells, for example in macrophages. Depending in the nature of interactions, the consequences can be inflammatory or anti-inflammatory. Also, the complement system can be activated on DAMPs or DAMP-like structures, which are generated by tissue injury, protein misfolding. or mislocalization. Such structures include, for example, mitochondria, membrane phospholipids, oxidized lipids in atherosclerosis, amyloid-β in brain, or oxidized bisretinoids on retinal pigment epithelial (RPE) cells in the macular area of the retina (64–66).

Polyunsaturated fatty acids are vulnerable to free radical attack caused by activated immune cells or cell apoptosis. As a consequence, oxidation-specific epitopes, such as oxidized phosphocholine, 4-hydroxynonenal, isolevuglandin, and malondialdehyde (MDA), are formed. The roles of oxidized lipids and MDA adducts in the pathogenesis of AMD, atherosclerosis and Alzheimer’s disease have been studied in more detail (67, 68). Interestingly, in AMD, factor H binds to both oxidized lipids and bisretinoids in drusen. Here, the common factor H variant 402Y has a higher affinity for oxidized lipids than the AMD risk allele 402H (69). Also the MDA-epitopes are recognized by factor H. Both the Y402H polymorphism in domain seven, and atypical hemolytic uremic syndrome (aHUS) associated mutations in domains 19–20 impair this interaction (70, 71). Therefore, the found association of factor H Y402H polymorphism with AMD, DDD and possibly also with atherosclerosis and Alzheimer’s disease could be related to the reduced binding of 402H to CRP and oxidized lipids compared to 402Y (19–21, 72). Reduced control of the amplification pathway of complement could thus contribute to the inflammatory pathology in these diseases.

Gangliosides are highly sialylated glycosphingolipids and abundantly expressed in the human body (73). Cells lacking terminal sialic acids, for example, due to oxidative damage, ischemic, senescent, necroptotic or apoptotic cell death become “DAMPs” for the complement system. Therefore, they serve as signals for complement activation and phagocytosis (2, 74). A distictive feature of the alternative pathway of complement is that its activators are structures that lack the ability to bind factor H (75). Thus, nearly any structure without suitable polyanions (sialic acids, glycosaminoglycans, and negatively charged phospholipids) or membrane complement regulators can act as complement activators as long as they provide binding sites for C3b molecules (76).

Because factor H interacts with cell surface sialic acids and surface deposited C3b, it has a crucial role in protecting self cells from complement attack. The presence of anti-ganglioside antibodies in atherosclerosis and Alzheimer’s disease patient sera has been previously observed (77, 78). It is therefore possible, although not shown, that these antibodies could interfere with factor H binding to sialic acids present on gangliosides and thereby contribute to inflammation. The antibodies could have a double-negative effect: They could activate the classical pathway and promote alternative pathway activation by preventing binding of factor H to cell surface gangliosides. A documented role for anti-ganglioside antibodies has been demonstrated in the neurological diseases Guillain-Barré and Miller Fisher syndromes [reviewed recently by Cutillo et al. (73)].

### Apolipoprotein E

Apolipoprotein E (ApoE) is the central molecule responsible for cholesterol metabolism in the liver, blood and brain. ApoE has been shown to be involved in amyloid-β clearance in the central nervous system. It promotes anti-atherosclerotic activity by regulating lipoprotein metabolism and promoting cholesterol efflux by the so called reverse cholesterol transport. ApoE also modulates macrophage polarization into the anti-inflammatory M2 phenotype (79, 80). ApoE has three allelic isoforms (apoE2/E3/E4) of which apoE4 is strongly associated with Alzheimer’s disease (81). ApoE4 is also associated with atherosclerosis, nephrotic glomerular disease in children and AMD, of which the latter has the strongest association with FH polymorphism Y402H (26, 81–85).

ApoE is found abundantly in the lipid-rich deposits of AMD, DDD, atherosclerosis and Alzheimer’s disease patients (12, 86–88). However, its function in plaque formation or clearance is not well known. *In vivo* apoE is mainly associated with lipids, such as HDL particles in plasma or with small HDL-like components in brain. However, a small portion of apoE is found in complex with lipid-free or lipid-poor proteins. Of these, especially the apoE4 variant is likely susceptible for self-aggregation and misfolding (89). Factor H binds both lipid-free and high density lipoprotein (HDL) associated apoE *via* domains 5–7 (4). On HDL particles factor H regulates the alternative pathway of complement but its role in binding to lipid-free apoE is not known. The single amino acid difference in residues 112 and 158 between the apoE isoforms is responsible for the structural differences between these proteins (90). Moreover, knowing that FH interacts with apoE *via* the domain 7, where the Y402H polymorphism is located, it is possible that these genetic variations could also affect binding between apoE and FH.

### Phagocytic Cells

Phagocytic cells are closely associated with disease progression in AMD, DDD, atherosclerosis, and Alzheimer’s disease. In atherosclerosis, macrophages play a crucial role in the phagocytosis of modified LDL particles and cholesterol efflux, while microglia are involved in amyloid-β phagocytosis. Increased intake of cholesterol by macrophages and reduced cholesterol efflux capacity leads to formation of foam cells in the arterial intima, while an increased intake of amyloid-β by microglial cells induces microglial activation and neurotoxicity (91). In microglia, the increase in free radical generation has been suggested to be related to the binding of amyloid-β to complement receptor type 3, CR3. CR3 is a phagocytic receptor that is involved in complement-mediated clearance of iC3b-coated particles and suppression of inflammation. It interacts with several different ligands, including factor H. Factor H interacts with CR3 on several cell types (92) and possibly has a direct effect on phagocytic functions. In cholesterol-loaded macrophages factor H has been shown to simultaneously promote cholesterol efflux, reduce transcription of proinflammatory genes and increase transcription of antiatherogenic genes such as ABCA1 and PPAR-α (93). PPAR-α transcription factor is known to induce the expression ABCA1, the intracellular ATP binding cassette transporter that regulates cellular cholesterol homeostasis (94). Binding of factor H to CR3 has been shown to reduce acute subretinal inflammation in mice indicating that factor H could be involved in suppressing inflammation in human AMD as well (95).

### Necrotic and Apoptotic Cells

Formation of necrotic and apoptotic cells is involved in the pathogenesis of AMD, C3G, atherosclerosis and Alzheimer’s disease. As mentioned earlier, complement system is involved in the non-inflammatory clearance of apoptotic and necrotic cells that is initiated through recognition of CRP by C1q that activates the classical pathway of complement (96, 97). Here, binding of factor H to CRP is crucial, as it is involved in suppressing activation of the alternative and terminal pathways at the site of tissue damage and during local inflammation (8, 98, 99).

While CR3 is the receptor of iC3b, complement receptor type 1 (CR1 and CD35) is the receptor that has a higher affinity toward C3b (100, 101). CR1 is a cell surface complement regulator that acts as a cofactor for factor I in the inactivation of C3b (similarly to factor H) and further in the cleavage of iC3b to C3c and C3dg. In addition to phagocytic cells CR1 is also found on red blood cells, where it participates in the clearance of immune complexes by transporting them for elimination in the spleen or liver (102, 103). Factor H blocks binding of CR1 to C3b (104) as it competes for the same binding site with CR1 on C3b. This competitive binding leads to decreased C3c/C3dg formation as only CR1, but not factor H, acts as a cofactor for factor I in the cleavage of iC3b to C3c and C3dg. As C3d/C3dg is recognized by CR2 on B-cells and thus links the innate and adaptive immunity, it is possible that inhibition of C3d formation by factor H could have consequences for the adaptive immunity and formation of autoantibodies that has been described at least in atherosclerosis and C3G (46, 105–107).

## Conclusions

AMD, C3G, atherosclerosis and Alzheimer’s disease seem to be unrelated to each other because of the different locations of the affected tissues. However, current knowledge on the role of complement-mediated inflammation in the development of these diseases indicates that they partially share common pathophysiology. Current genetic and biochemical data indicate that complement regulator factor H participates in the modification of both complement activation and cell responses and that defects in complement regulation by factor H play an important role in the pathogenesis of these diseases. More knowledge is, however, needed to understand the exact molecular mechanisms whereby factor H protects the eyes, the kidneys, the brain, and arteries from inflammation that eventually leads to formation of lipid-rich deposits in AMD, C3G, Alzheimer’s disease, and atherosclerosis.

## Author Contributions

SM and KH have been involved with original research leading to concepts described in this work. They both wrote the review. All authors contributed to the article and approved the submitted version.

## Funding

The study was supported by the Jane and Aatos Erkko Foundation (15032019), Finnish Foundation for Cardiovascular Research (08042019), Academy of Finland (331108), and Helsinki University Hospital Funds (VTR).

## Conflict of Interest

The authors declare that the research was conducted in the absence of any commercial or financial relationships that could be construed as a potential conflict of interest.
